# Development of a Blocking ELISA Based on a Monoclonal Antibody against a Predominant Epitope in Non-Structural Protein 3B2 of Foot-and-Mouth Disease Virus for Differentiating Infected from Vaccinated Animals

**DOI:** 10.1371/journal.pone.0111737

**Published:** 2014-11-04

**Authors:** Yuanfang Fu, Zengjun Lu, Pinghua Li, Yimei Cao, Pu Sun, Meina Tian, Na Wang, Huifang Bao, Xingwen Bai, Dong Li, Yingli Chen, Zaixin Liu

**Affiliations:** State Key Laboratory of Veterinary Etiological Biology, National Foot-and-Mouth Disease Reference Laboratory, Lanzhou Veterinary Research Institute, Chinese Academy of Agriculture Science, Lanzhou, Gansu, China; Institut National de la Santé et de la Recherche Médicale U 872, France

## Abstract

A monoclonal antibody (McAb) against non-structural protein (NSP) 3B of foot-mouth-disease virus (FMDV) (3B4B1) was generated and shown to recognize a conserved epitope spanning amino acids 24–32 of 3B (GPYAGPMER) by peptide screening ELISA. This epitope was further shown to be a unique and predominant B cell epitope in 3B2, as sera from animals infected with different serotypes of FMDV blocked the ability of McAb 3B4B1 to bind to NSP 2C3AB. Also, a polyclonal antibody against NSP 2C was produced in a rabbit vaccinated with 2C epitope regions expressed in *E. coli*. Using McAb 3B4B1 and the 2C polyclonal antibody, a solid-phase blocking ELISA (SPB-ELISA) was developed for the detection of antibodies against NSP 2C3AB to distinguish FMDV-infected from vaccinated animals (DIVA test). The parameters for this SPB-ELISA were established by screening panels of sera of different origins. Serum samples with a percent inhibition (PI) greater than or equal to 46% were considered to be from infected animals, and a PI lower than 46% was considered to indicate a non-infected animal. This test showed a similar performance as the commercially available PrioCHECK NS ELISA. This is the first description of the conserved and predominant GPYAGPMER epitope of 3B and also the first report of a DIVA test for FMDV NSP 3B based on a McAb against this epitope.

## Introduction

Foot-and-mouth disease (FMD) is a highly contagious and economically devastating viral disease of cloven-hoofed animals. The causative agent, foot-and-mouth disease virus (FMDV), has a positive sense and single-stranded RNA genome of 8400 nucleotides that codes for 12 proteins. VP1–4 are structural proteins (SPs) that make up the capsid of the virus, which is the main component of the inactivated vaccine. L, 2A, 2B, 2C, 3A, 3B, 3C, and 3D are non-structural proteins (NSPs) that participate in viral replication and play additional roles within the host cell [1]. Differentiation of FMDV-infected animals from inactivated vaccine-inoculated animals (DIVA) mainly depends on the detection of antibodies against NSPs because most of these proteins are removed from the vaccine by some kind of purification method during production. Therefore, suboptimal vaccine purity may affect diagnostic specificity, as the presence of NSPs in some vaccine preparations may result in misclassification of animals that have been repeatedly vaccinated [2]. The FMDV antigen used to formulate the vaccine needs to be purified to reduce the NSP content so that the vaccine does not induce antibodies to NSPs. This effort is necessary in countries wishing to be recognized as FMD-free with vaccination. However, purification of the vaccine antigen increases the cost of production. Therefore, identification of conserved and immunodominant B cell epitopes in the NSPs of FMDV will provide a basis for the development of epitope-deleted marker vaccines and corresponding DIVA tests, which will significantly improve the efficiency of disease control programs.

Numerous FMDV-specific linear B cell epitopes have been identified in FMDV NSPs, including in 2B, 2C, 3A, 3B, and 3D [3]. Several DIVA tests have been developed based on detection of antibodies against these predominant B cell epitopes. One research group identified a linear B cell epitope (CELHEKVSSHPIFKQ) in 2C and developed a synthetic peptide ELISA based on this epitope [4]; they subsequently developed an epitope-blocking ELISA for DIVA diagnosis that uses a monoclonal antibody (McAb) targeting the core repeat epitope QKPLK in NSP 3B [5]. A recent study also described a competition ELISA using a McAb against an immunodominant linear epitope (MRKTKLAPTVAHGVF) in NSP 3D [6]. The same group then mutated the above epitope in 3D and the core repeat epitope QKPLK in 3B to develop a negative marker vaccine with improved DIVA capabilities [7]. The FMDV NSP 3B exists as three similar but non-identical tandem repeats, 3B1 (VPg1), 3B2 (VPg2), and 3B3 (VPg3), that share a high amino acid sequence homology and a common core amino acid motif [QKPL (M) K] [3]. These proteins were found to have a high density of linear B cell epitopes, some of which, including GPYAGPMERQKPLK, PMERQKPLKVKAKA, and QKPLKVKAKAPVVK, are conserved and immunodominant and could potentially be used to develop DIVA tests [8].

In this study, we identified a McAb, 3B4B1, that recognizes a conserved and immunodominant epitope (^24^GPYAGPMER^32^) located in 3B2 of NSP 3B. Because 3B2 can be deleted without significantly affecting the replication of FMDV [9], we developed a solid-phase blocking ELISA (SPB-ELISA) using 3B4B1 as a competitive antibody and 2C3AB NSP produced in *E. coli* as the antigen [10]. This research provides a useful tool for DIVA diagnosis of FMD and a potential companion DIVA test for a negative marker vaccine with deleted 3B2.

## Materials and Methods

### Ethics statement

Before the beginning of the experiment, all animals were acclimatized for one week. Rabbits and mice for preparation of antibodies were bred in clean and spacious animal rooms. Swine, bovine and sheep for FMDV infection were raised in bio-safety level 3 (BSL-3) containment facility in Lanzhou Veterinary Research Institute (LVRI). All animals were handled humanely according to the rules described by the Animal Ethics Procedures and Guidelines of the People's Republic of China, and the study was approved by the Animal Ethics Committee of LVRI, Chinese Academy of Agricultural Sciences (Permit No. LVRIAEC2010-006). All animals used in the present study were humanely bred and bled. Swine, bovine and sheep were euthanized by exsanguination under deep anesthesia (intramuscular injection of chlorpromazine at 2–6 mg/kg) at the end of the experiment.

### Preparation of Recombinant Proteins

The expression and purification of the NSPs 3A, 3B, 2C epitope region, 3D, 3ABC, and 2C3AB of FMDV were carried out according to previously described methods [10], [11].

### Production of Polyclonal Antibody against 2C Epitope Regions

The purified 2C epitope region was emulsified in Montanide ISA 206 adjuvant (Seppic, Paris, France) and used to immunize rabbits to produce specific polyclonal antibodies. The rabbit was immunized hypodermically with 200 µg/0.5 ml of 2C epitope protein vaccine three times at 2 week intervals. One week after the third injection, rabbits were bled to collect the sera. The 2C antibody titers were determined by an indirect ELISA using 2C3AB as the coating antigen. The polyclonal antibodies against 2C epitope region protein were purified from the sera of the immunized rabbits using an Affi-Gel protein G column (GE healthcare, OH, USA) and stored at −20°C for later use.

### Production of Monoclonal Antibody (McAb) against 3B

McAbs were produced according to traditional protocols [12]. Briefly, female BALB/c mice were immunized three times with 100 µg of purified 3B protein emulsified in Montanide ISA 206 adjuvant (Seppic, Paris, France) at 2 week intervals. After 2 weeks, the mice were boosted intraperitoneally with 100 µg of purified protein without adjuvant. The mice were subsequently sacrificed 3 days after the final vaccination. The spleen cells of mice were fused with SP2/0 cells. After 2 weeks, supernatants from the hybridomas were screened using recombinant 3B in an indirect ELISA. Horseradish peroxidase (HRP)-conjugated McAb was prepared with the EZ-Link Plus Activated Peroxidase kit (Thermo, USA). The reactivity of McAbs to different recombinant NSPs was determined by indirect ELISA according to the procedure described previously [11].

### Peptide ELISA Screening for Binding Epitopes of the McAbs

Six peptides were synthesized by Genscript Inc. (Nanjin, China) based on the complete amino acid sequence of 3B from FMDV O/CHA/99 (Table 1). The purity of these peptides was determined by HPLC to be ≥90%. The peptide ELISA was performed according to the method described by Hohlich et al. [3] to analyze the binding epitopes of different McAbs against 3B. Briefly, microtiter plates (Corning, Salt Lake City, USA) were coated with synthetic peptides. After blocking, hybridoma supernatants containing the McAbs were added to each plate. After washing, HRP-conjugated goat anti-mouse IgG (Sigma, USA) was added and TMB (3, 3′, 5, 5′-tetramethyl-benzidine) substrate (SurModics, USA) was used for color development. The optical density (OD) was measured at 450 nm using an automated plate reader (Bio-Rad, USA).

**Table 1:** Synthetic peptides used to identify the FMDV-specific B cell epitope recognized by the McAbs.

Peptide no.	Location	Amino acid sequence
Pep 1	1–14	GPYTGPLERQKPLK
Pep 2	10–23	QKPLKVRAKLPQQE
Pep 3	24–37	GPYAGPMERQKPLK
Pep 4	29–42	PMERQKPLKVKVKA
Pep 5	43–56	PVVKEGPYEGPVKK
Pep 6	57–71	PVALKVKAKNLIVTE

### Selection of the McAb Binding to a Native Epitope of NSP 3B

To determine whether or not the McAbs bound to a native epitope of NSP 3B, a blocking ELISA was performed by coating microtiter plates with recombinant NSP 2C3AB (3 µg/mL) in coating buffer overnight at 4°C. After three washes with PBST (0.01 M PBS and 0.05% Tween-20), test sera from FMDV-infected (positive control) or non-infected (negative control) cattle diluted 1∶5 with serum dilution buffer (2.5 g/L casein and 10% equine serum in PBS, pH 7.2) were added to the plate and incubated at 37°C for 1 h. After washing, serially diluted McAbs were added and incubated for 1 h at 37°C. After washing, an HRP-conjugated goat anti-mouse antibody was added to the wells and the plate was incubated at 37°C for 1 h. Color development and OD readings were done as described above. The ratios of OD values of negative to positive serum (N/P) were calculated. A greater N/P ratio indicates that sera from infected cattle have a greater ability to block the binding of the McAbs to recombinant FMDV NSP 2C3AB.

### Conservation of B Cell Epitopes in 3B Protein Among Different FMDV Serotypes

The homology of 3B amino acid sequences was analyzed by alignment of sequences from different serotypes of FMDV. The selected reference virus strains and corresponding GenBank numbers were O/Tibet/CHA/99 (CAD62370.1), O/Akesu/58 (AAM44304.1), O/TAW/99 (CAD62208.1), O/TAW/99 (AAT01778.1), Asia 1/JS/CHA/2005 (ABM66095.1), Asia1/YS/CHA/2005 (ADU56664.1), Asia1/VN/LC04/2005 (ADC92544.1), A/IRQ/09 (AER28328.1), A/VIT/2004 (AEO16200.1), and A22/Iraq/64 (AAT01706.1). The amino acid sequences were aligned using DNASTAR Lasergene 7.1 software (DNASTAR, Inc., Madison, WI, USA).

### Serum Samples of Different Origins

The following serum samples were used in this study: (1) sera from healthy, unvaccinated animals, including 152, 123, and 162 serum samples from cattle, sheep, and swine, respectively, which were found to have no antibodies (<1∶4) against O or Asia 1 FMDV by liquid-phase blocking ELISA (LPBE); (2) sera from clinically healthy, vaccinated animals, including 24 serum samples from cattle and 48 serum samples from swine that had been inoculated twice with inactivated vaccine for type O/CHA/99 FMDV (PD_50_>6.0); (3) sera from infected animals, including 121 serum samples from cattle infected with Asia 1/JS/CHA/05 or O/CHA/99 FMDV at 10–28 days postinfection (DPI), 40 serum samples from four cattle infected with A/WH/CHA/2009 FMDV at 0–229 DPI, 48 serum samples from two sheep infected with O/CHA/99 FMDV at 0–417 DPI, four serum samples from four sheep infected with Asia 1/JS/05 at 28 DPI, nine serum samples from nine pigs infected with Asia 1/JS/05 at 16–28 DPI, 38 serum samples from 38 pigs infected with O/CHA/99 at 11–60 DPI, and 16 serum samples from one pig infected with O/CHA/99 FMDV at 0–194 DPI; (4) field sera, including 200 serum samples collected from cattle in FMDV-endemic regions of China in 2009; and (5) control sera, including positive control serum samples derived from cattle, sheep, and swine infected with the O/CHA/99 strain of FMDV at 30 DPI, weakly positive serum samples derived from animals that developed a low-level antibody titer against both SP and NSP 3ABC 15 days after infection with Asia 1/JS/05 FMDV, and negative control serum samples derived from clinically healthy, unvaccinated animals.

### Solid-Phase Blocking ELISA

The solid-phase blocking ELISA (SPB-ELISA) was performed according to the protocol of Sørensen et al. [13], with some modifications. Briefly, ELISA plates were coated with 2.0 µg/ml purified 2C polyclonal antibody diluted in carbonate buffer (pH 9.6) in a 100 µL volume and incubated overnight at 4°C. After three washes with PBST, the plates were sealed with 10 mg/ml gelatin and incubated for 45 min at 37°C. After three washes with PBST, purified 2C3AB protein was diluted to an optimal concentration in PBST, 100 µl/well was added, and the plate was incubated for 60 min at 37°C. After five washes, serum samples were diluted 1∶5 in dilution buffer (PBS containing 0.25% casein, 10% horse serum, and 3% *E. coli* lysate), 100 µl/well was added, and the plate was incubated on a plate rocker overnight at room temperature (20–25°C). Following washing, the optimal dilution of HRP-conjugated McAb in dilution buffer was added, and the plate was incubated at 37°C for 1 h. Color development and OD readings were done as described in section 2.4. The percent inhibition (PI) of the sample was derived according to the following formula: PI = (1−test sample OD/negative control OD)×100%.

### Parameters of the SPB-ELISA

Parameters for the SPB-ELISA were established using panels of sera of different origins: sera from uninfected cattle (n = 152), sheep (n = 123), and swine (n = 162); and sera from infected cattle (n = 121), sheep (n = 52), and swine (n = 63). The PI for each sample was calculated. The cutoff PI value was selected based on the frequency distribution at different PIs to result in relatively high sensitivity and specificity. Diagnostic sensitivity was calculated as the proportion of the infected herd that tested positive, and diagnostic specificity was calculated as the proportion of vaccinated or non-vaccinated healthy animals that tested negative.

### Validation of the SPB-ELISA

The performance of the SPB-ELISA was determined by comparison with a 3ABC ELISA [14] and the PrioCHECK NS ELISA (Prionics AG, Schlieren-Zurich, Switzerland) using a test panel of sera of different origins. Samples included 61 serum samples from cattle infected with O/CHA/99 or Asia 1/JS/05 FMDV at 10–28 DPI; 40 serum samples from swine infected with O/CHA/99 or Asia 1/JS/05 FMDV at 0–194 DPI; 41 serum samples from two sheep infected with O/CHA/99 FMDV at 0–417 DPI; 50 and 43 sera from healthy cattle and swine, respectively, that were determined to be antibody-negative against O and Asia 1 FMDV by LPBE; 24 serum samples from clinically healthy cattle aged 1–2 years; 48 serum samples from swine that had been vaccinated twice with inactivated vaccine, taken at 14 DPI; 200 serum samples from field cattle herds; 40 serum samples from four cattle infected experimentally with A/WH/CHA/2009 at 0–229 DPI; and 16 serum samples from one pig infected with O/CHA/99 at 0–194 DPI.

The 3ABC-ELISA antibody titer was expressed as the ratio of the experimental sample to the positive control. In this assay, a cutoff value of 0.2 has been reported to result in relatively high sensitivity and specificity [14]. The PrioCHECK NS ELISA (Prionics AG, Schlieren-Zurich, Switzerland) is a commercial blocking ELISA kit that can be applied to all susceptible animal species. In this test, the antibody titer was expressed as the PI compared with the negative control. A PI of 50% is the threshold for qualitative judgment of infection.

## Results

### Titer of the 2C Polyclonal Antibody

Polyclonal antibodies against the 2C protein were collected from the blood of immunized rabbits. The optimal concentration of the purified 2C polyclonal antibody for use as a coating antibody was determined to be 2.0 µg/ml in a capture ELISA using recombinant NSP 2C3AB as the antigen. This concentration was chosen for use in the SPB-ELISA.

### Characteristics of McAbs

**Figure 1:**
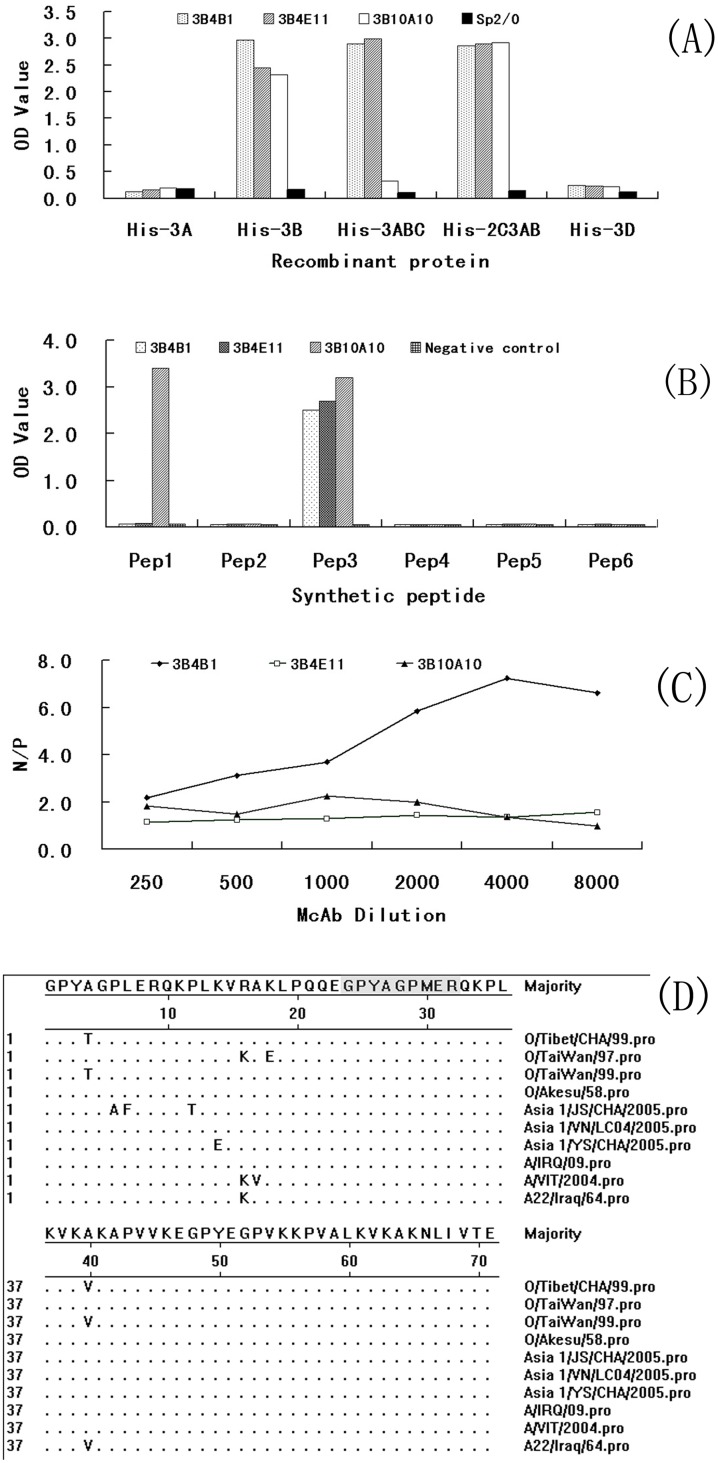
Characteristics of the monoclonal antibodies. (A) The reactivity of the McAbs with various recombinant non-structural proteins. (B) Identification of the epitopes recognized by the McAbs against NSP 3B by peptide ELISA. (C) The N/P values of the McAbs at different dilutions of serum. (D) Conservation of the motifs in FMDV NSP 3B.

Three McAbs, 3B4B1, 3B4E11, and 3B10A10, were screened. McAbs 3B4B1 and 3B4E11 were classified as IgG1, and 3B10A10 was classified as IgG2b using a commercially available isotype classification kit. All three McAbs specifically bind to recombinant proteins His-3B, His-3ABC, and His-2C3AB, but not His-3A or His-3D, as determined by an indirect ELISA (Fig. 1A), indicating that the three McAbs recognize epitopes located within protein 3B. A peptide screening ELISA showed that 3B4B1 and 3B4E11 only react with peptide 3, not with other peptides containing the previously reported QKPLK core motif [7], indicating that 3B4B1 and 3B4E11 specifically bind to the ^24^GPYAGPMER^32^ epitope. McAb 3B10A10 reacted with peptide 3 and with peptide 1 (Fig. 1B), indicating that it might recognize a space epitope.

The McAb recognizing a native epitope in NSP 2C3AB was selected by using sera from FMDV-infected cattle to block the binding of the three McAbs to NSP 2C3AB, as described in the Materials and Methods. As shown in Figure 1C, only the N/P value of McAb 3B4B1 was greater than 2.1 at different serum dilutions, indicating that McAb 3B4B1 recognized the native epitope ^24^GPYAGPMER^32^ located in NSP 3B2.

### Conservation of the Epitopes in FMDV 3B Protein

Sequence alignment showed that the epitope^ 1^GPYTGPLER^9^ in 3B1 was not conserved among different serotypes of FMDV; however, the epitope ^24^GPYAGPMER^32^ in 3B2 was well-conserved in the O, A, and Asia 1 serotypes of FMDV (Fig. 1D).

### The Cutoff Value, Sensitivity, and Specificity of the SPB-ELISA

**Figure 2:**
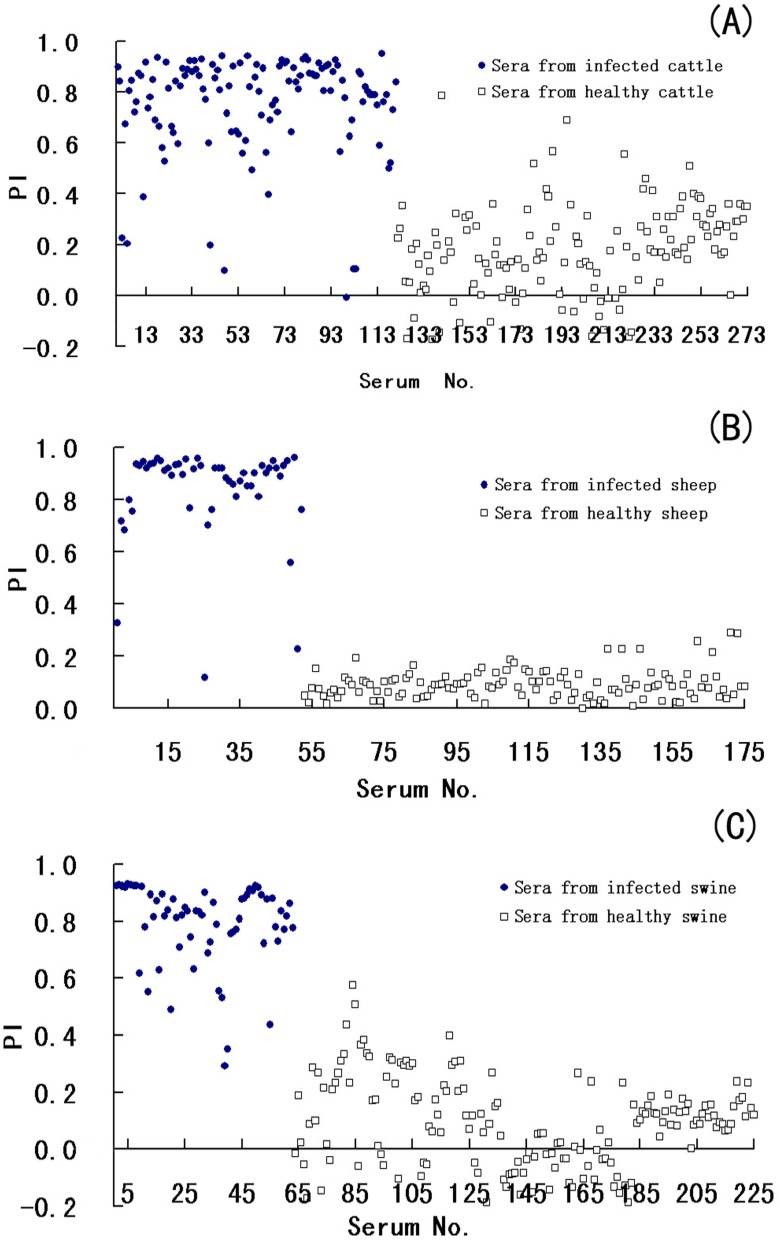
Frequency distribution of PI values obtained by SPB-ELISA. (A) Sera from cattle, (B) sera from sheep, and (C) sera from swine.

The cutoff PI value for the SPB-ELISA was determined by testing 437 serum samples from healthy unvaccinated animals and 236 serum samples from infected animals. The frequency distributions of the resultant PI values are shown in Figure 2A–C. Table 2 shows the specificity and sensitivity of the assay at three different cutoff values, 40%, 46%, and 50%. Using a cutoff value of 46%, the sensitivity and specificity of the assay were relatively high in the three animal species. Based on these data, serum samples with a PI equal to or greater than 46% were considered to be from infected animals, and samples with a PI lower than 46% were considered to be from non-infected animals. As a quality control measure taken in this assay, the PIs of the positive and weakly positive controls were required to be greater than 70% and 50%, respectively.

**Table 2:** Specificity and sensitivity of the SPB-ELISA at different cutoff values.

		Specificity	Specificity	Sensitivity
Species	Cutoff value	Naive animals	Vaccinated animals	Infected animals
Cattle	40%	92.1% (140/152)	91.7% (22/24)	92.6% (112/121)
	46%	95.4% (145/152)	95.8% (23/24)	92.6% (112/121)
	50%	95.4% (145/152)	95.8% (23/24)	91.7% (111/121)
Swine	40%	97.5% (158/162)	97.9% (47/48)	96.8% (61/63)
	46%	98.8% (160/162)	100% (48/48)	95.2% (60/63)
	50%	98.8% (160/162)	100% (48/48)	93.7% (59/63)
Sheep	40%	100% (122/122)	–	94.2% (49/52)
	46%	100% (122/122)	–	94.2% (49/52)
	50%	100% (122/122)	–	94.2% (49/52)

Using the cutoff value described above, specificities of 95.4% (145/152), 100% (123/123), and 98.8% (160/162) and sensitivities of 92.6% (112/121), 94.2% (49/52), and 95.2% (60/63) were obtained for non-infected and infected cattle, sheep, and swine sera, respectively. These results demonstrate that most animals infected with FMDV develop antibodies against the corresponding epitope of McAb 3B4B1. Thus, the McAb 3B4B1 binding epitope ^24^GPYAGPMER^32^ is a predominant B cell epitope in NSP 3B2 of FMDV.

### Comparison of the SPB-ELISA with two other ELISAs

As shown in Table 3, for 61 sera from infected cattle, 59, 60, and 56 samples were positive by SPB-ELISA, 3ABC-ELISA, and PrioCHECK NS ELISA, respectively. For 200 sera from field cattle, 22, 19, and 17 samples were positive by SPB-ELISA, 3ABC-ELISA, and PrioCHECK NS ELISA, respectively. For 40 sera from infected swine, 36, 36, and 37 samples were positive by SPB-ELISA, 3ABC-ELISA, and PrioCHECK FMDV-NS ELISA, respectively, and for 41 sera from infected sheep, 39, 35, and 38 samples were positive. For sera from uninfected animals, all three methods gave negative results, except the 3ABC-ELISA, which gave one positive result from a vaccinated swine. The coincident rates between the SPB-ELISA and the 3ABC-ELISA and between the SPB-ELISA and the PrioCHECK NS ELISA were 95.1% (482/507) and 95.7% (462/483), respectively.

**Table 3:** Comparison of the concordance rates between the SPB-ELISA and the 3ABC-ELISA or the PrioCHECK FMDV-NS ELISA.

		SPB-ELISA		3ABC-ELISA(no. of coincident)		PrioCHECK NSP(no. of coincident)	
Origin of sera	Total no.	Positive	Negative	Positive	Negative	Positive	Negative
Infected cattle^a^	61	59	2	60 (56)	1 (0)	56 (55)	5 (0)
Infected swine^b^	40	36	4	36 (34)	4 (1)	37 (35)	3 (1)
Infected sheep^c^	41	39	2	35 (35)	6 (2)	38 (37)	3 (1)
Vaccinated cattle	24	0	24	0	24 (24)	/	/
Vaccinated swine	48	0	48	1 (0)	47 (47)	0	48 (48)
Non-infected cattle	50	0	50	0	50 (50)	0	50 (50)
Non-infected swine	43	0	43	0	43 (43)	0	43 (43)
Field cattle	200	22	178	19 (16)	181 (174)	17 (16)	183 (176)
Total no.	507	156	351	151 (141)	356 (341)	148 (143)	335 (319)

a Sera from cattle infected with O/CHA/99 or Asia 1/JS/05 FMDV at 10–28 DPI.

b Sera from swine infected with O/CHA/99 or Asia 1/JS/05 FMDV at 0–194 DPI.

c Sera from sheep infected with O/CHA/99 FMDV at 0–417 DPI.

**Figure 3:**
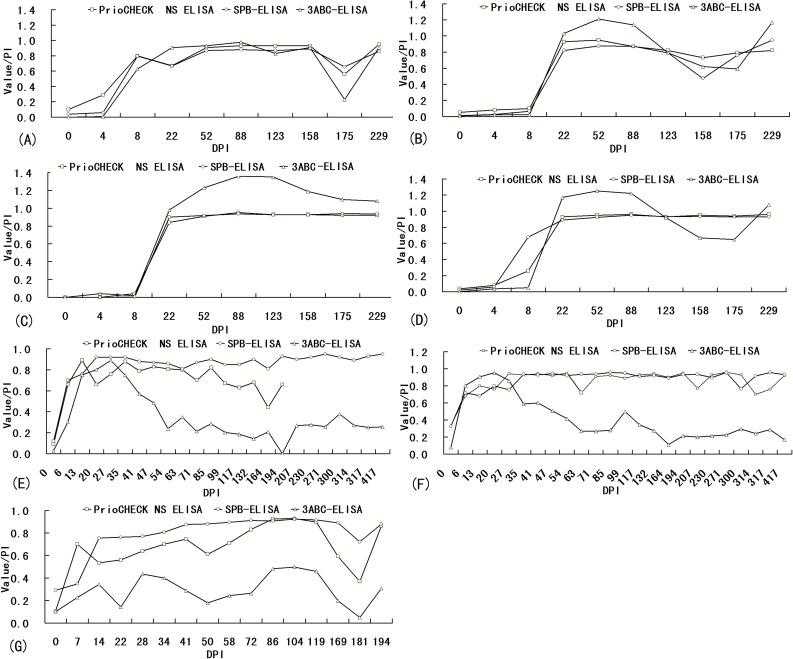
Detection of antibodies by SPB-ELISA and two commercial ELISA kits (3ABC-ELISA and PrioCHECK FMDV-NS ELISA) in sera collected sequentially from animals infected with FMDV. (A)–(D) show the results from cattle no. 4009, 0738, 4017, and 4004, respectively, (E) and (F) show the results from sheep no. 54 and 40, and (G) shows the result from pig no. 39.

Results from 97 sequential serum samples from four cattle (n = 40), two sheep (n = 41), and one swine (n = 16) infected with FMDV are shown in Figure 3A–G. Of the 40 serum samples from four infected cattle, 39 samples yielded identical results by the SPB-ELISA, 3ABC-ELISA, and PrioCHECK NS ELISA. Only one sample, taken from animal no. 4004 at 8 DPI, showed a positive result by SPB-ELISA (Fig. 3D). Of the 41 serum samples from two infected sheep, only one differential result, taken from animal no. 54 at 164 DPI, was observed between the SPB-ELISA and the PrioCHECK NS ELISA (Fig. 3E); however, differential results were observed between the 3ABC-ELISA and the other two ELISAs in four samples: the sample taken from sheep no. 40 at 164 DPI (Fig. 3F), and the samples taken from sheep no. 54 at 117, 132, and 194 DPI (Fig. 3E). Of the 16 sera from an infected swine, one negative result at 7 DPI by the SPB-ELISA, one negative result at 181 DPI by the PrioCHECK NS ELISA, and three negative results, at 22, 50, and 181 DPI, by the 3ABC-ELISA were observed (Fig. 3G).

## Discussion

FMD is the most important viral infectious disease of livestock. Some ruminants can become virus carriers after infection even though a vaccination strategy has been adopted. Differentiation of naturally infected from vaccinated animals is necessary to evaluate the effects of disease control measures and to detect early signs of FMD incursion or transmission in epidemic regions. The development of a negative marker vaccine and its corresponding DIVA test will improve the efficacy of disease control strategies without the increased production costs of highly purified vaccines. Identification and screening of conserved and immunodominant epitopes are critically important for the development of negative marker vaccines and the corresponding DIVA tests. In this study, three McAbs against 3B NSP were prepared using prokaryotically expressed 3B protein. One McAb, 3B4B1, was determined to specifically bind to a conserved and immunodominant epitope of 3B2, ^24^GPYAGPMER^32^, which is distinct from the ^1^GPYTGPLER^14^ epitope of 3B1 and the ^48^GPYEGPVKK^56^ epitope of 3B3. It seems that ^27^A and ^30^M are critical residues influencing the binding capacity of McAb 3B4B1 to the epitope ^24^GPYAGPMER^32^. Moreover, McAb 3B4B1 also bound to one native and immunodominant B cell epitope in NSP 2C3AB, and this binding capacity could be blocked by serum samples from FMDV-infected animals. A previous study indicated that 3B1, 3B2, and 3B3 tandem repeats share a common core motif, QKPL(M)K, which is the core motif of several B cell epitopes [3]. Animals infected with a genetically modified FMDV with a mutated core motif did not produce antibodies to the above motif [7], indicating that native B cell epitopes also exist within the core motif regions. Here, we show that ^24^GPYAGPMER^32^ in 3B2 is another unique and native B cell epitope that is well-conserved among different serotypes of FMDV and can be mutated to abolish binding by the corresponding McAb (unpublished data). These results provide more information on B cell epitopes in FMDV NSPs and provide a basis for the development of a negative marker vaccine against FMD.

To develop a blocking ELISA for the detection of an antibody response to the identified epitope of McAb 3B4B1, NSP 2C3AB protein, which contains the major B cell epitope regions of 2C and the entire 3AB region of the NSP of FMDV [10], [11], was used as the antigen. In addition, 2C polyclonal antibody produced in rabbits was used as a capture antibody, and McAb 3B4B1 was used as a detection antibody to develop a SPB-ELISA test for DIVA purposes. The cutoff PI value of this SPB-ELISA was determined to be 46% by screening panels of sera of different origins. Based on this threshold, the sensitivity of the assay was 92.6% in cattle, 95.2% in swine, and 94.2% in sheep, and the specificity for non-infected animals was 95.4% in cattle, 98.8% in swine, and 100% in sheep. When compared with two commercial ELISA kits, the 3ABC-ELISA and the PrioCHECK NS ELISA, the coincident rate of the SPB-ELISA with the PrioCHECK NS ELISA (95.7%) was higher than that of the SPB-ELISA with the 3ABC-ELISA (95.1%), indicating that the SPB-ELISA has a similar performance rate as the PrioCHECK NS ELISA and a higher specificity than the indirect 3ABC-ELISA.

Over the past 2 decades, detection of NSP antibodies has been accepted as a reliable method for evaluating the infectious status of animal herds with or without vaccination [13]–[17]. The accuracy of DIVA tests can be compromised by interference from NSPs remaining in the vaccine [16]. In South America, a combined system of an indirect ELISA (3ABC-ELISA) with an enzyme-linked immunoelectrotransfer blot assay was successfully used to support local FMD control programs under systematic vaccination [15]. However, development of a negative marker vaccine will provide an improved strategy to overcome the above difficulties.

In conclusion, a McAb target against the immunodominant epitope, ^24^GPYAGPMER^32^_,_ located in 3B2, was identified and used to develop a DIVA test for FMDV surveillance. The ELISA described here is a potential companion DIVA method for an FMDV negative marker vaccine, which will greatly improve the efficacy of FMD control and eradication efforts in the future.
